# Editorial: Editor’s challenge: Abhishek Mahajan - how can precision oncology be advanced with validated imaging-based nomograms?

**DOI:** 10.3389/fonc.2024.1362187

**Published:** 2024-02-01

**Authors:** Abhishek Mahajan, Shreya Shukla, Richa Vaish, Manish Devendra Mair

**Affiliations:** ^1^ Department of Imaging, The Clatterbridge Cancer Centre National Health Service (NHS) Foundation Trust, Liverpool, United Kingdom; ^2^ Faculty of Health and Life Sciences, University of Liverpool, Liverpool, United Kingdom; ^3^ Department of Radio Diagnosis, Tata Memorial Hospital, Varanasi, India; ^4^ Department of Head and Neck Surgical Oncology, Tata Memorial Hospital, Mumbai, India; ^5^ Department of Maxillofacial Surgery, University Hospitals of Leicester National Health Service (NHS) Trust, Leicester, United Kingdom

**Keywords:** precision oncology, precision medicine, radiomics, artificial intelligence, nomogram, imaging

## Introduction

Every individual undergoing cancer treatment, along with the unique characteristics of their cancer, presents an array of data points that can pave the way for a targeted strategy to halt the progression of that specific tumor. In the past, deciphering this molecular information was hindered by its high cost and time-intensive nature. Fortunately, recent advancements in data collection and interpretation have brought us to the brink of harnessing this wealth of information to tailor therapies on an individual basis. This knowledge gave birth to the concept of precision oncology. Today, we are poised to utilize the distinct biological features of tumor to counteract its growth.

While precision oncology traditionally emphasized the cancer genome, broadening the scope to include RNA and proteins, opens up possibilities for discovering novel options in targeted treatments. The integration of real-world data, notably from the rapidly expanding pool of electronic health records, is now actively deployed to aid in evaluating the effectiveness of various treatment approaches. Recently, there has been a significant rise in the identification of additional molecular targets that impact various facets of cancer.

Translational research serves as the vital link between fundamental scientific discoveries and its application in clinical settings, leading to impactful health outcomes. Imaging stands at the core of this continuum, facilitating the translation of molecular discoveries into practical clinical use. Concurrently, pertinent clinical observations spark scientific inquiries, propelling advancements in research (1).

Over the past decade, the field of medical image analysis has experienced exponential growth. This expansion is marked by an augmented array of pattern recognition tools and an escalation in the sizes of datasets. These strides have enabled the establishment of high-throughput procedures for extracting quantitative features, transforming images into data that can be mined, and subsequently analyzing this data for decision support. This evolving practice is commonly referred to as radiomics. While radiomics holds applicability across a broad spectrum of medical conditions, its most advanced state of development is observed in oncology (2).

Radiomics presents a vast array of imaging biomarkers, seemingly limitless in scope, with the potential to play a crucial role in various aspects of cancer management. These biomarkers can contribute to cancer detection, diagnosis, prognostic evaluation, prediction of treatment response, and monitoring of disease status.

Nomograms serve as a visual representation of intricate mathematical formulas. In the realm of medicine, these graphical tools utilize biological, clinical and radiological variables to visually illustrate a statistical prognostic model. This model generates the probability of a specific outcome based on the unique characteristics of the variables on an individual basis (3).

Precision oncology can be significantly advanced through the integration of validated imaging-based nomograms. There are several ways in which this synergy can enhance cancer care: Personalized Treatment Strategies, Prognostic Assessment, Response Prediction, Risk Stratification, Clinical Trial Design and Patient Selection, Monitoring Treatment Response and Enhanced Communication. In essence, the integration of validated imaging-based nomograms into precision oncology practices strengthens the ability to make informed, individualized decisions at every stage of cancer management, from diagnosis to treatment planning and ongoing monitoring.

Advancements in Precision Oncology are attributable to progress in Magnetic Resonance Imaging (MRI), Computed Tomography (CT), ultrasound, Positron Emission Tomography (PET), radiogenomics, Artificial Intelligence (AI), and optical imaging (4, 5). The rapid development of highly flexible AI models is poised to revolutionize medicine. The Generalist Medical Artificial Intelligence (GMAI) is capable of diverse tasks with minimal task-specific data (6). The use of comprehensive structured synoptic templates in reporting promotes clear communication, reduces the omission of crucial information, and ensures the inclusion of essential details for optimal individualized management planning, contributing to precision oncology (7).

Led by Dr. Mahajan and a dedicated team, this Research Topic delves into the diverse ways and domains where the integration of validated imaging-based nomograms can propel the field of precision oncology forward. The Research Topic explores how these nomograms can be strategically applied to enhance the precision, effectiveness, and personalized nature of oncological practices. The Research Topic consists of seven manuscripts, each of which is an original research study. Below is a summary of each of these contributions.

In their original research study, Kobatake et al. explore the prognostic significance of radiological morphology (RM) across all stages and to elucidate the molecular characteristics that differentiate each type of RM in patients diagnosed with clear cell renal cell carcinoma (ccRCC). They have come up with the idea of a novel treatment strategy for clear cell renal cell carcinoma (ccRCC) with heightened radiological morphology complexity by the specific targeting of the ubiquitin-proteasome system.

Sarcopenia is linked to reduced survival and heightened complications in individuals with renal cell carcinoma. The ability to promptly identify patients with low muscle composition, who might face worse outcomes or could benefit from preoperative intervention, holds clinical significance. Schmeusser et al., have explored linear segmentation, a practical technique for assessing muscle composition. This method enables the integration of muscle composition analysis into clinical decision-making.

The original research by Li et al. aims to formulate and validate the efficacy of an unenhanced magnetic resonance imaging (MRI)-based integrated radiomics nomogram for the discrimination between low-grade and high-grade chondrosarcoma.

Qiu et al. have investigated the predictive potential of gadoxetic acid-enhanced magnetic resonance imaging (MRI), coupled with T1 mapping and clinical factors, in determining Ki-67 expression in hepatocellular carcinoma (HCC).

The prognosis of patients with tumor deposits (TDs) in locally advanced rectal cancers is similar or worse than that of patients with metastatic lymph nodes. The primary objective of the study by Lv et al. is to detect TDs by MRI and evaluate its predictive value.

Tong et al. have successfully developed and validated a prediction model that integrates radiomics features and N stage. This model proves effective in predicting the four-year recurrence risk among patients with esophageal squamous cell carcinoma (ESCC) who undergo surgery.

Li et al. have conducted an analysis of the clinical and ultrasound characteristics of breast sclerosing adenosis (SA) and invasive ductal carcinoma (IDC). Furthermore, they have formulated a predictive nomogram for sclerosing adenosis based on their findings.

## Conclusion

The editors express sincere gratitude to all the authors, reviewers, and members of the editorial board for their valuable contributions to this Research Topic. It is our hope that this Research Topic will serve as inspiration for future and innovative research approaches in the realm of precision oncology and radiomics in oncology.

## Author contributions

AM: Conceptualization, Data curation, Formal analysis, Funding acquisition, Investigation, Methodology, Project administration, Resources, Software, Supervision, Validation, Visualization, Writing – original draft, Writing – review & editing. SS: Conceptualization, Data curation, Formal analysis, Funding acquisition, Investigation, Methodology, Project administration, Resources, Software, Supervision, Validation, Visualization, Writing – original draft, Writing – review & editing. RV: Writing – original draft, Writing – review & editing, Conceptualization, Data curation, Formal analysis, Funding acquisition, Investigation, Methodology, Project administration, Resources, Software, Supervision, Validation, Visualization. MM: Writing – original draft, Writing – review & editing, Conceptualization, Data curation, Formal analysis, Funding acquisition, Investigation, Methodology, Project administration, Resources, Software, Supervision, Validation, Visualization.

## References

[1] Mahajan A Goh V Basu S Vaish R Weeks AJ Thakur MH. Bench to bedside molecular functional imaging in translational cancer medicine: to image or to imagine? Clin Radiol (2015) 70(10):1060–82. 6. doi: 10.1016/j.crad.2015.06.082 26187890

[2] Gillies RJ Kinahan PE Hricak H. Radiomics: images are more than pictures, they are data. Radiology. (2016) 278(2):563–77. doi: 10.1148/radiol.2015151169 PMC4734157 26579733

[3] Balachandran VP Gonen M Smith JJ DeMatteo RP. Nomograms in oncology: more than meets the eye. Lancet Oncol (2015) 16(4):e173–80. doi: 10.1016/S1470-2045(14)71116-7 PMC4465353 25846097

[4] Vaidya T Agrawal A Mahajan S Thakur MH Mahajan A. The continuing evolution of molecular functional imaging in clinical oncology: the road to precision medicine and radiogenomics (Part I). Mol diagnosis Ther (2019) 23(1):1–26. doi: 10.1007/s40291-018-0366-4 30411216

[5] Vaidya T Agrawal A Mahajan S Thakur MH Mahajan A. The continuing evolution of molecular functional imaging in clinical oncology: the road to precision medicine and radiogenomics (Part II). Mol Diagnosis Ther (2019) 23(1):27–51. doi: 10.1007/s40291-018-0367-3 30411216

[6] Chakrabarty N Mahajan A. Imaging analytics using artificial intelligence in oncology: A comprehensive review. Clin Oncol (R Coll Radiol) (2023) 27:S0936–6555(23)00334-5. doi: 10.1016/j.clon.2023.09.013 37806795

[7] Mahajan A Chakrabarty N Majithia J Ahuja A Agarwal U Suryavanshi S. Multisystem imaging recommendations/guidelines: in the pursuit of precision oncology. Indian J Med Paediatric Oncol (2023) 44(01):002–25. doi: 10.1055/s-0043-1761266

